# Effect of educational lecture on the diagnostic accuracy of Japan NBI Expert Team classification for colorectal lesions

**DOI:** 10.1186/s12876-021-01676-x

**Published:** 2021-03-05

**Authors:** Yuki Okamoto, Shiro Oka, Shinji Tanaka, Yuki Kamigaichi, Hirosato Tamari, Yasutsugu Shimohara, Tomoyuki Nishimura, Katsuaki Inagaki, Hidenori Tanaka, Kenta Matsumoto, Ken Yamashita, Kyoku Sumimoto, Yuki Ninomiya, Nana Hayashi, Yasuhiko Kitadai, Kenichi Yoshimura, Kazuaki Chayama

**Affiliations:** 1 Department of Gastroenterology and Metabolism, Hiroshima University Hospital, 1-2-3, Kasumi, Minami-ku, Hiroshima, 734-8551 Japan; 2 Department of Endoscopy, Hiroshima University Hospital, Hiroshima, Japan; 3 Department of Health Sciences, Prefectural University of Hiroshima, Hiroshima, Japan; 4 Center for Integrated Medical Research, Hiroshima University Hospital, Hiroshima, Japan

**Keywords:** Colorectal tumor, Education, Endoscopic diagnosis, Japan NBI Expert Team classification (JNET classification), Narrow-band imaging (NBI)

## Abstract

**Background:**

An educational and training program is required for generalization of Japan NBI Expert Team (JNET) classification. However, there is no detailed report on the learning curve of the diagnostic accuracy of endoscopists using JNET classification. We examined the effect of an educational lecture on beginners and less experienced endoscopists for improving their diagnostic accuracy of colorectal lesions by JNET classification.

**Methods:**

Seven beginners with no endoscopy experience (NEE group), 7 less experienced endoscopists (LEE group), and 3 highly experienced endoscopists (HEE group) performed diagnosis using JNET classification for randomized NBI images of colorectal lesions from 180 cases (Type 1: 22 cases, Type 2A: 105 cases, Type 2B: 33 cases, and Type 3: 20 cases). Next, the NEE and LEE groups received a lecture on JNET classification, and all 3 groups repeated the diagnostic process. We compared the correct diagnosis rate and interobserver agreement before and after the lecture comprehensively and for each JNET type.

**Results:**

In the HEE group, the correct diagnosis rate was more than 90% with good interobserver agreements (kappa value: 0.78–0.85). In the NEE and LEE groups, the correct diagnosis rate (NEE: 60.2 → 68.0%, *P* < 0.01; LEE: 66.4 → 86.7%, *P* < 0.01), high-confidence correct diagnosis rate (NEE: 19.6 → 37.2%, *P* < 0.01; LEE: 43.6 → 61.1%, *P* < 0.01), and interobserver agreement (kappa value, NEE: 0.32 → 0.43; LEE: 0.39 → 0.75) improved after the lecture. In the examination by each JNET type, the specificity and positive predictive value in the NEE and LEE groups generally improved after the lecture.

**Conclusion:**

After conducting an appropriate lecture, the diagnostic ability using JNET classification was improved in beginners or endoscopists with less experience in NBI magnifying endoscopy.

## Introduction

**Fig. 1:**
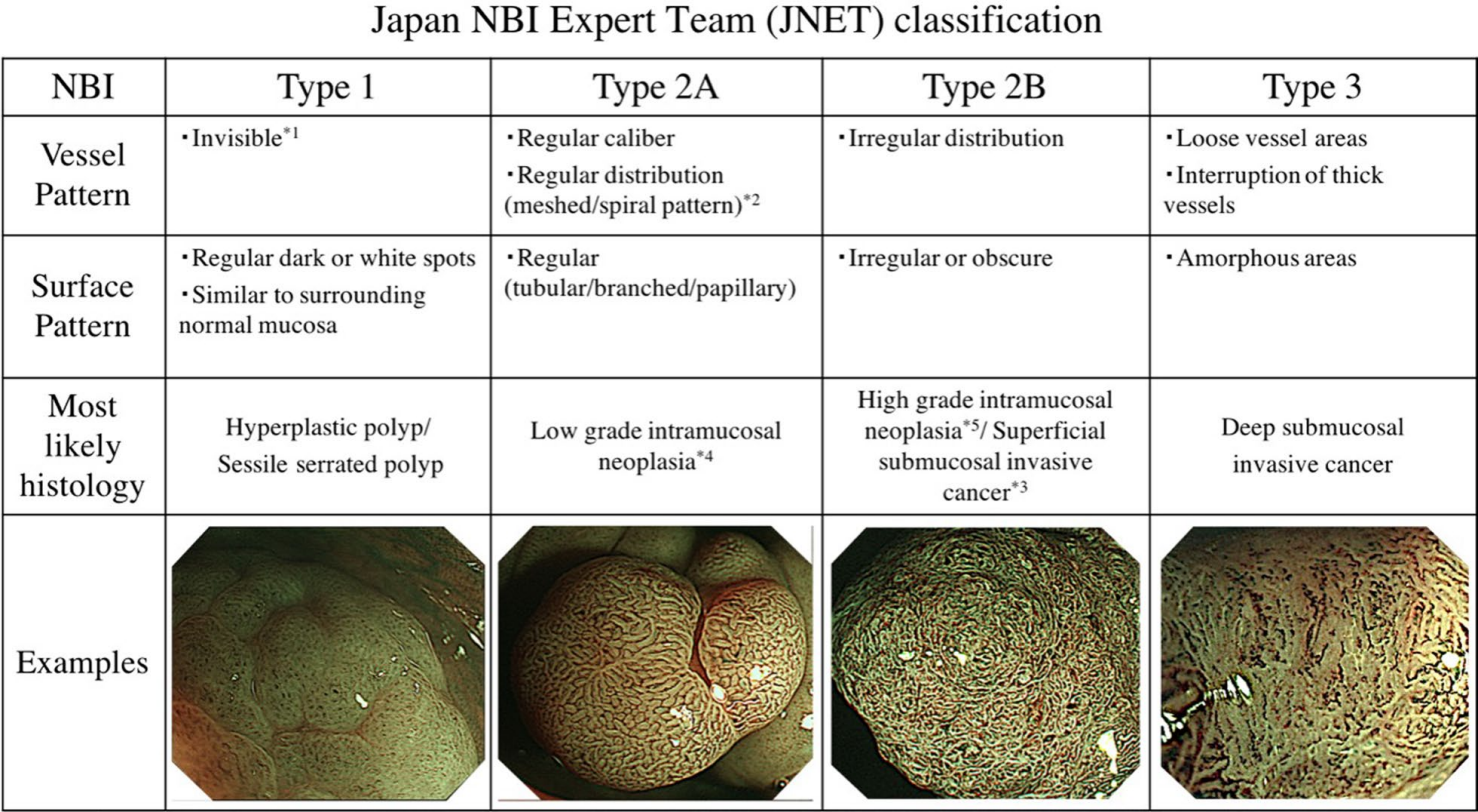
Japan NBI Expert Team (JNET) Classification details. *1. If visible, the caliber in the lesion is similar to the surrounding normal mucosa. *2. Microvessels are often distributed in a punctate pattern and well-ordered reticular or spiral vessels may not be observed in depressed lesions. *3. Deep submucosal invasive cancer may be included. *4. Low grade intramucosal neoplasia: low grade dysplasia. *5. High grade intramucosal neoplasia: high grade dysplasia

Narrow-band imaging (NBI), introduced in 2006, is one of the commonly applied endoscopic image enhancement methods. It has become a popular tool in general clinical use for the endoscopic diagnosis of colorectal lesions. In Japan, NBI magnifying colonoscopy had been used for the diagnosis of colorectal lesions under various classifications [1–4]; however, the coexistence of multiple terms for same or similar findings resulted in confusion. In 2009, the NBI International Colorectal Endoscopic (NICE) classification was proposed by the Colon Tumor NBI Interest Group [5]. The NICE classification is simple and widely used in countries where the application of magnifying colonoscopy is not extensive [6, 7]. However, with NICE classification, it is difficult to distinguish between low grade dysplasia (LGD) to superficial submucosal invasive (SM-s) carcinoma. In 2014, based on the NICE classification, the Japan NBI Expert Team (JNET) proposed a unified NBI magnifying endoscopy classification, the JNET classification (Fig. 1) [8]. The JNET classification consists of Type 1, 2A, 2B and 3 based on vessel/surface pattern. Each type refers to the most likely histology of the colorectal lesions. Type 1 is characterized by an invisible vessel pattern and a surface pattern with dark and white spots or similar to the surrounding normal mucosa. Type 1 includes hyperplastic polyp (HP) and sessile serrated polyp (SSP). Type 2A is characterized by a regular vessel pattern, such as regular caliber or distribution, and a regular surface pattern. Type 2A includes LGD. Type 2B is characterized by an irregular vessel pattern, such as a variable caliber or irregular distribution, and an irregular to obscure surface pattern. Type 2B includes high grade dysplasia (HGD) and SM-s carcinoma. Type 3 is characterized by loose vessel areas and/or interruption of thick vessels and/or an amorphous surface pattern. Type 3 includes deep submucosal invasive (SM-d) carcinoma [8]. In the validation studies conducted thus far, Types 1, 2A, and 3 are highly reliable diagnostic indicators with high specificity for each expected histology [9–14]. Follow-up, endoscopic treatment, and surgical resection are recommended for Type 1, Type 2A, and Type 3 lesions, respectively. However, Type 2B includes a wide variety of lesions ranging from HGD to SM-d carcinoma; therefore, pit pattern diagnosis with chromoendoscopy is necessary for an accurate diagnosis. In previous papers, JNET classification was reported to be useful even for colonoscopists with little experience in NBI magnifying endoscopy [15–18]. However, it was pointed out that appropriate training is necessary to improve the diagnostic accuracy using JNET classification [16].

Although it is acknowledged that education on NBI diagnosis is useful in the differentiation of neoplastic and non-neoplastic lesions [19–25], there is no detailed report on the learning curve of the diagnostic accuracy using JNET classification. In this study, we examined the diagnostic accuracy of JNET classification among diagnosticians with various experience levels of NBI magnifying endoscopy before and after an educational lecture on JNET classification.

## Materials and methods

### Image for NBI diagnosis

The images were captured with NBI using high-definition colonoscopes (CF-H260AZI; Olympus, Tokyo, Japan) and a standard videoendoscopic system (EVIS LUCERA ELITE system; Olympus, Tokyo, Japan). The NBI endoscopic images of 180 consecutive cases were used to conduct the test: 22 cases of Type 1, 105 cases of Type 2A, 33 cases of Type 2B, and 20 cases of Type 3 lesions (Table 1). The histopathological diagnosis of each case was made by two pathologists at the Hiroshima University Hospital. The pathological characteristics of each JNET type lesions were as follows: Type 1 included HP (4 cases: 18.2%), SSP (17 cases: 77.3%), and LGD (1 case: 4.5%); Type 2A included LGD (86 cases: 81.9%), HGD (18 cases: 17.1%), and SM-s carcinoma (1 case: 1.0%); Type 2B included LGD (7 cases: 21.2%), HGD (9 cases: 27.3%), SM-s carcinoma (3 cases: 9.1%), and SM-d carcinoma (14 cases: 42.4%); Type 3 included HGD (1 case: 5.0%) and SM-d carcinoma (19 cases: 95.0%). In this study, the relationship between JNET classification results and pathological findings is similar to a previous report on the examination of a total of 2933 cases except for Type 2B lesion: Type 1 included HP/SSP (119 cases: 98%) and LGD (3 cases: 2%); Type 2A included HP/SSP (17 cases: 1%), LGD (1626 cases: 86%), HGD (230 cases: 12%), and SM-s carcinoma (15 cases: 1%); Type 2B included LGD (297 cases: 37%), HGD (340 cases: 43%), SM-s carcinoma (67 cases: 8%), and SM-d carcinoma (95 cases: 12%); Type 3 included HGD (1 case: 1%), SM-s carcinoma (5 cases: 4%), and SM-d carcinoma (118 cases: 95%) [9]. Therefore, in this study, the correct answer for JNET classification was considered as the gold standard.

**Table 1:** Clinicopathological features of the cases for diagnostic endoscopic images

Variables	NBI Diagnosis by JNET classification				
	Type 1 [n = 22]	Type 2A [n = 105]	Type 2B [n = 33]	Type 3 [n = 20]	Total [n = 180] (%)
Tumor size (mm, mean ± SD)	21.0 ± 11.7	22.2 ± 13.0	27.0 ± 15.5	19.8 ± 7.6	22.8 ± 13.1
*Tumor location*					
Right colon	16 (72.7)	44 (41.9)	13 (39.4)	6 (30.0)	80 (44.4)
Left colon	6 (27.3)	27 (25.7)	10 (30.3)	6 (30.0)	49 (27.2)
Rectum	0 (0)	34 (32.4)	10 (30.3)	8 (40.0)	51 (28.3)
*Gross type*					
Superficial	18 (81.8)	27 (25.7)	11 (33.3)	11 (55.0)	70 (38.9)
Polypoid	4 (18.2)	78 (74.3)	22 (66.7)	9 (45.0)	110 (61.1)
*Histology*					
Hyperplastic	4 (18.2)	0 (0)	0 (0)	0 (0)	4 (2.2)
SSP	17 (77.3)	0 (0)	0 (0)	0 (0)	17 (9.5)
LGD	1 (4.5)	86 (81.9)	7 (21.2)	0 (0)	94 (52.2)
HGD	0 (0)	18 (17.1)	9 (27.3)	1 (5.0)	28 (15.6)
SM-s carcinoma	0 (0)	1 (1.0)	3 (9.1)	0 (0)	4 (2.2)
SM-d carcinoma	0 (0)	0 (0)	14 (42.4)	19 (95.0)	33 (18.3)

*JNET* Japan NBI Expert Team, *HP* hyperplastic lesion, *SSP* sessile serrated polyp, *LGD* low-grade dysplasia, *HGD* high-grade dysplasia, *SM-s carcinoma* superficial submucosal invasive carcinoma (< 1000 mm), *SM-d carcinoma* deep submucosal invasive carcinoma (≧ 1000 mm)

The correct diagnosis using JNET classification was determined by an expert endoscopist who is a member of the JNET working group (S.T.) with an excellent intra-observer agreement (kappa value: 1.0). The diagnostic images before and after the lecture on JNET classification were randomly sorted. In addition, the correct answer was withheld from the evaluators until the end of the study.

### Image evaluator categories

**Fig. 2:**
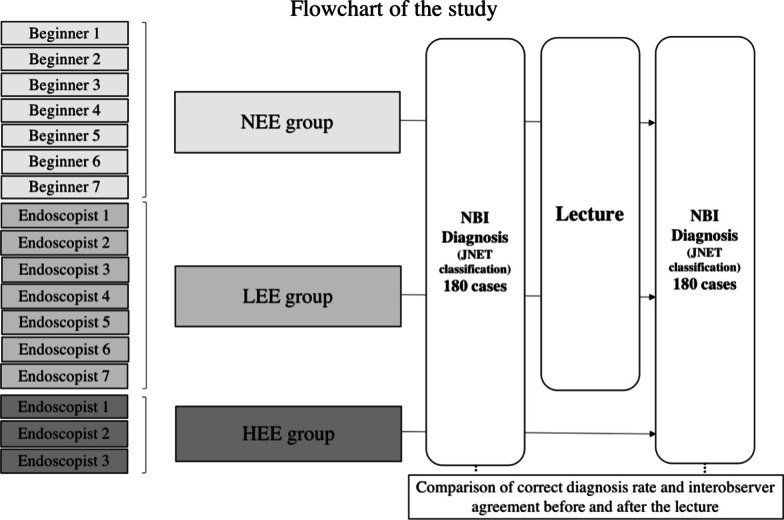
Flow chart of the study. *NEE* beginners with no prior endoscopy experience, *LEE* less experienced endoscopists, *HEE* highly experienced endoscopists, *NBI* narrow-band imaging, *JNET* Japan NBI Expert Team

The evaluators were divided into 3 groups: 7 beginners (4 medical students and 3 initial residents) without the experience of endoscopic diagnosis (the no endoscopy experience group, NEE group), 7 endoscopists with an experience of < 5 years in endoscopic diagnosis using magnifying NBI (the less experienced endoscopist group, LEE group), and 3 endoscopy specialists with an experience of > 5 years in endoscopic diagnosis using magnifying NBI (the highly experienced endoscopist group, HEE group) (Fig. 2).

### Educational lecture on JNET classification

Prior to the first NBI diagnosis test, we explained the outline concept of JNET classification using Fig. 1 to the NEE/LEE groups for 5 min. Next, members of the NEE/LEE/HEE groups took the first NBI diagnosis test. After the first test, an educational lecture was imparted to the NEE/LEE groups. The lecture was based on the definition of JNET classification with 12 concrete images representing each type of lesion and reflecting the images presented in the annotations of Fig. 1 (Type 1, Type 2A, Type 2B, and Type3: 3, 3, 4, and 2 images, respectively). In addition, we presented 6 images for distinguishing Type 1 and 2A, Type 2A and 2B, and Type 2B and 3. After the participants diagnosed the images, we presented the correct answer and explained the points of the diagnosis. The lecturer was an endoscopist familiar with NBI and JNET classification with the same experience level as that of the HEE group. The lecture was conducted using a predetermined phrase for 15 min while presenting the images on electronic slides. Five hours after the lecture, members of the NEE/LEE/HEE groups took the second NBI diagnosis test. Both tests, before and after the lecture, and the lecture were given on the same day.

### Statistical analysis

The correct diagnosis rate and high-confidence correct diagnosis rate were calculated for the total tests before and after the lecture in the NEE, LEE, and HEE groups. In addition, the accuracy rate, sensitivity, specificity, positive predictive value (PPV), and negative predictive value (NPV) for the tests of each JNET type were calculated.

The correct diagnosis rate of the total tests and the accuracy rate, sensitivity, and specificity of the tests of each JNET type were compared before and after the lecture using the McNemar test. The PPV and NPV of the tests of each JNET type were compared before and after the lecture using the Pearson’s chi-squared test. *P* < 0.05 was considered statistically significant. The statistical analyses were performed using JMP version 14 (SAS Institute Inc., Cary, NC, USA). The interobserver agreement in each group was assessed using kappa values as follows: < 0.4: poor agreement; 0.41–0.60: fair agreement; 0.61–0.80: good agreement; > 0.8: excellent agreement. Kappa statistics were calculated using the Fleiss method of R version 3.6.0 (R Core Team (2019). R: A language and environment for statistical computing. R Foundation for Statistical Computing, Vienna, Austria. URL https://www.R-project.org/).

## Results

### Diagnostic performance of all JNET types

In the NEE group, the correct diagnosis rate (60.2% → 68.0%, *P* < 0.01), the high-confidence correct diagnosis rate (19.6% → 37.2%, *P* < 0.01), and the interobserver agreement (kappa value: 0.32 → 0.43) significantly improved after the lecture (Table 2). In the LEE group, the correct diagnosis rate (66.4% → 86.7%, *P* < 0.01), the high-confidence correct diagnosis rate (43.6% → 61.1%, *P* < 0.01), and the interobserver agreement (kappa value: 0.39 → 0.75) significantly improved after the lecture. In the HEE group, the correct diagnosis rate was 90.6–92.0%, and the high-confidence correct diagnosis rate was 80.6–83.5%. The kappa value of the HEE group was 0.78–0.85.

**Table 2:** Correct diagnosis rate and correct diagnosis rate with high confidence intervals

Group	Correct diagnosis rate	
	Correct diagnosis rate with high confidence	
	[kappa value]	
	Before the lecture	After the lecture*
NEE	60.2 (57.5–62.9)	68.0 (65.4–70.6)^+^
	19.6 (17.4–21.8)	37.2 (34.6–40.0)^+^
	[0.32]	[0.43]
LEE	66.4 (63.8–69.0)	86.7 (84.8–88.5)^+^
	43.6 (40.8–46.3)	61.1 (58.4–63.8)^+^
	[0.39]	[0.75]
HEE	90.6 (88.1–93.9)	92.0 (89.8–94.3)
	80.6 (77.2–83.9)	83.5 (80.4–86.6)^+^
	[0.85]	[0.78]

(95% confidence interval)

^*^HEE group has no lecture

^+^Denotes statistical significance *P* < 0.01

### Diagnostic performance of JNET type 1

The sensitivity, specificity, PPV, NPV, and accuracy rate in the NEE group were 73.4%, 97.0%, 77.4%, 96.3%, and 94.1% before the lecture and 89.0%, 97.6%, 83.5%, 98.4%, and 96.5% after the lecture, respectively (Table 3). The sensitivity, NPV, and accuracy rate significantly increased after the lecture. The interobserver agreements in the NEE group were poor before and after the lecture (0.0408 → 0.128).

**Table 3:** Diagnostic performance of NBI for JNET Type 1 lesions before and after the lecture

Variables	NEE		LEE		HEE*	
	Before	After	Before	After	Before	After
Sensitivity	73.4 (67.9–78.0)	89.0 (84.5–92.4)^+^	77.3 (75.0–77.3)	97.4 (94.6–98.9)	100 (97.1–100)	100 (97.1–100)
Specificity	97.0 (96.3–97.7)	97.6 (96.9–98.0)	100 (99.7–100)	99.3 (98.9–99.5)	100 (99.6–100)	100 (99.6–100)
PPV	77.4 (71.7–82.2)	83.5 (79.3–86.7)	100 (97.1–100)	94.9 (92.2–96.4)^++^	100 (97.1–100)	100 (97.1–100)
NPV	96.3 (95.6–97.0)	98.4 (97.8–98.9)^+^	96.9 (96.6–96.9)	99.6 (99.2–99.8)^+^	100 (99.6–100)	100 (99.6–100)
Accuracy	94.1 (92.8–95.2)	96.5 (95.4–97.3)^+^	77.2 (96.7–97.2)	99.0 (98.4–99.4)^+^	100 (99.3–100)	100 (99.3–100)
κ-Value	0.0408	0.128	− 0.00525	0.0651	1.0	1.0

*JNET* Japan NBI Expert Team

(95% confidence interval)

^*^HEE group has no lecture

^+^Denotes statistical significance *P* < 0.01

^++^Denotes statistical significance *P* < 0.05

The sensitivity, specificity, PPV, NPV, and accuracy rate in the LEE group were 77.3%, 100%, 100%, 96.9%, and 77.2% before the lecture and 97.4%, 99.3%, 94.9%, 99.6%, and 99.0% after the lecture, respectively. The PPV, NPV, and accuracy rate significantly increased after the lecture. The interobserver agreements in the LEE group were poor before and after the lecture (− 0.00525 → 0.0651).

The sensitivity, specificity, PPV, NPV, and accuracy rate in the HEE group were all 100%.

### Diagnostic performance of JNET type 2A

The sensitivity, specificity, PPV, NPV, and accuracy rate in the NEE group were 57.4%, 91.6%, 90.6%, 60.6%, and 71.7% before the lecture and 63.9%, 94.5%, 94.2%, 65.2%, and 76.7% after the lecture, respectively (Table 4). The sensitivity, PPV, and accuracy rate significantly increased after the lecture. The interobserver agreements in the NEE group were poor before and after the lecture (0.126 → 0.112).

**Table 4:** Diagnostic performance of NBI for JNET Type 2A lesions before and after the lecture

Variables	NEE		LEE		HEE*	
	Before	After	Before	After	Before	After
Sensitivity	57.4 (55.7–58.8)	63.9 (62.5–65.1)^+^	63.7 (61.9–65.2)	86.0 (85.0–86.6)^+^	86.3 (85.2–86.3)	90.2 (88.7–96.6)^++^
Specificity	91.6 (89.3–93.6)	94.5 (92.4–96.1)	89.3 (86.8–91.5)	98.1 (96.7–98.9)^+^	100 (98.5–100)	99.1 (97.1–99.8)
PPV	90.6 (87.9–92.7)	94.2 (92.0–95.8)^++^	89.3 (86.8–91.5)	98.4 (97.3–99.1)^+^	100 (98.7–100)	99.3 (97.7–99.8)
NPV	60.6 (59.0–61.9)	65.2 (63.8–66.3)	63.7 (61.9–65.2)	83.3 (82.1–84.1)^+^	84.0 (82.7–84.0)	87.8 (86.0–88.4)
Accuracy	71.7 (69.7–73.3)	76.7 (75.0–78.0)^+^	74.4 (72.3–76.1)	91.0 (89.9–91.7)^+^	92.0 (90.7–92.0)	93.9 (92.2–94.4)^++^
κ-Value	0.126	0.112	0.13	0.357	0.461	0.0697

*JNET* Japan NBI Expert Team

(95% confidence interval)

^*^HEE group has no lecture

^+^Denotes statistical significance *P* < 0.01

^++^Denotes statistical significance *P* < 0.05

The sensitivity, specificity, PPV, NPV, and accuracy rate in the LEE group were 63.7%, 89.3%, 89.3%, 63.7%, and 74.4% before the lecture and 86.0%, 98.1%, 98.4%, 83.3%, and 91.0% after the lecture, respectively. All parameters in the LEE group significantly increased after the lecture. The interobserver agreements in the LEE group were poor before and after the lecture (0.13 → 0.357).

The sensitivity, specificity, PPV, NPV, and accuracy rate in the HEE group were 86.3–90.2%, 99.1–100%, 99.3–100%, 84.0–87.8%, and 92.0–93.9%, respectively, and the interobserver agreements were poor to fair (0.0697–0.461).

### Diagnostic performance of JNET type 2B

The sensitivity, specificity, PPV, NPV, and accuracy rate in the NEE group were 59.3%, 68.5%, 29.7%, 88.2%, and 66.8% before the lecture and 63.6%, 73.8%, 35.3%, 90.0%, and 71.9% after the lecture, respectively (Table 5). The specificity and accuracy rate significantly increased after the lecture. The interobserver agreements in the NEE group were poor before and after the lecture (0.0589 → 0.0705).

**Table 5:** Diagnostic performance of NBI for JNET Type 2B lesions before and after the lecture

Variables	NEE		LEE		HEE*	
	Before	After	Before	After	Before	After
Sensitivity	59.3 (53.6–64.8)	63.6 (58.0–69.0)	68.8 (63.2–74.0)	86.6 (82.2–90.1)^+^	93.9 (88.2–97.1)	91.9 (86.0–95.7)
Specificity	68.5 (67.2–69.8)	73.8 (72.5–75.0)^+^	69.5 (68.2–70.6)	87.7 (86.7–88.4)^+^	89.8 (88.5–90.5)	92.1 (90.7–92.9)^++^
PPV	29.7 (26.8–32.5)	35.3 (32.1–38.2)	33.6 (30.9–36.1)	61.2 (58.1–63.6)^+^	67.4 (63.3–69.7)	72.2 (67.5–75.2)
NPV	88.2 (86.6–89.8)	90.0 (88.5–91.5)	90.9 (89.2–92.4)	96.7 (95.6–97.5)^+^	98.5 (97.1–99.3)	98.1 (96.6–99.0)
Accuracy	66.8 (64.7–68.9)	71.9 (69.8–73.9)^+^	69.4 (67.3–71.3)	87.5 (85.9–88.8)^+^	90.6 (88.5–91.7)	92.0 (89.9–93.4)
κ-Value	0.0589	0.0705	0.00736	0.144	0.29	− 0.0703

*JNET* Japan NBI Expert Team

(95% confidence interval)

^*^HEE group has no lecture

^+^Denotes statistical significance *P* < 0.01

^++^Denotes statistical significance *P* < 0.05

The sensitivity, specificity, PPV, NPV, and accuracy rate in the LEE group were 68.8%, 69.5%, 33.6%, 90.9%, and 69.4% before the lecture and 86.6%, 87.7%, 61.2%, 96.7%, and 87.5% after the lecture, respectively. All parameters in the LEE group significantly increased after the lecture. The interobserver agreements in the LEE group were poor before and after the lecture (0.00736 → 0.144).

The sensitivity, specificity, PPV, NPV, and accuracy rate in the HEE group were 91.9–93.9%, 89.8–92.1%, 67.4–72.2%, 98.1–98.5%, and 90.6–92.0%, respectively, and the interobserver agreements were poor (− 0.0703 to 0.29).

### Diagnostic performance of JNET type 3

The sensitivity, specificity, PPV, NPV, and accuracy rate in the NEE group were 62.9%, 91.0%, 46.6%, 95.1%, and 87.9% before the lecture and 73.6%, 93.1%, 57.2%, 96.6%, and 91.0% after the lecture, respectively (Table 6). The sensitivity, specificity, PPV, and accuracy rate significantly increased after the lecture. The interobserver agreements in the NEE group were poor before and after the lecture (0.031 → 0.192). The sensitivity, specificity, PPV, NPV, and accuracy rate in the LEE group were 65.0%, 95.3%, 63.2%, 95.6%, and 91.9% before the lecture and77.9%, 97.9%, 82.6%, 97.3%, and 95.7% after the lecture, respectively. All parameters in the LEE group were significantly increased after than that before the lecture. The interobserver agreements in the LEE group were poor before and after the lecture (0.246 → 0.268). The sensitivity, specificity, PPV, NPV, and accuracy rate of the HEE group were 93.3–96.7%, 98.8%, 90.3–90.6%, and 99.2–99.6%, and 98.1–98.5%, respectively, and the interobserver agreements were poor (− 0.0714 to − 0.0345).

**Table 6:** Diagnostic performance of NBI for JNET Type 3 lesions before and after the lecture

Variables	NEE		LEE		HEE*	
	Before	After	Before	After	Before	After
Sensitivity	62.9 (55.8–69.4)	73.6 (67.0–79.3)^++^	65.0 (58.5–70.8)	77.9 (72.6–82.1)^+^	96.7 (90.8–99.0)	93.3 (86.8–96.9)
Specificity	91.0 (90.1–91.8)	93.1 (92.3–93.8)^++^	95.3 (94.5–96.0)	97.9 (97.3–82.1)^+^	98.8 (98.0–99.0)	98.8 (97.9–99.2)
PPV	46.6 (41.3–51.4)	57.2 (52.1–61.7)^++^	63.2 (56.9–68.9)	82.6 (76.9–87.1)^+^	90.6 (85.1–92.8)	90.3 (84.0–93.8)
NPV	95.1 (94.2–96.0)	96.6 (95.7–97.3)	95.6 (94.8–96.3)	97.3 (96.6–97.8)^++^	99.6 (98.8–99.9)	99,2 (98.3–99.6)
Accuracy	87.9 (86.3–89.3)	91.0 (89.5–92.2)^+^	91.9 (90.5–93.2)	95.7 (94.5–96.7)^+^	98.5 (97.2–99.0)	98.1 (96.7–98.9)
κ-Value	0.031	0.192	0.246	0.268	− 0.0345	− 0.0714

*JNET* Japan NBI Expert Team

(95% confidence interval)

^*^HEE group has no lecture

^+^Denotes statistical significance *P* < 0.01

^++^Denotes statistical significance *P* < 0.05

## Discussion

The clinical usefulness of JNET classification has been examined, and the description of Types 1, 2A, and 3 is considered useful for not only experts but also inexperienced evaluators in diagnosing the histological type or invasion depth of a colorectal tumor [9–18]. However, the educational effects and learning curves remain unclear. In this study, the pre-lecture diagnostic performance for JNET classification in the NEE and LEE groups was similar to that of previous studies [15, 16, 18]. Even in the NEE group, it was possible to make correct diagnosis of typical cases to some extent before the lecture. Therefore, JNET classification is useful for beginners.

In addition, a short educational lecture on JNET classification resulted in an improvement in diagnostic ability and interobserver agreement in the NEE and LEE groups. In the HEE group, high sensitivity and specificity were obtained for all types. In addition to having a large number of NBI diagnoses, members in the HEE group have discussed cases using the JNET classification during a daily endoscopic procedure or in case conferences at our institution for at least 3 years. This practice may result in a good diagnostic ability using the JNET classification.

With regard to Type 1 cases, the specificity in the NEE and LEE groups was as good as 95% or more before and after the lecture. Therefore, it is considered there is little risk that cases requiring resection will not be treated. On the other hand, the sensitivity before the lecture was low in the NEE and LEE groups. Comparing images that were misdiagnosed by more than half of each group with other images (Additional file 1: Fig. 3), most of the misdiagnosed images presented visible vessel patterns. It was considered that the misdiagnosis was caused by the misunderstanding of the following wordings in the JNET classification chart: if the vessel pattern is visible, the honeycomb pattern and caliber in the lesion is similar to that of the surrounding normal mucosa.

The specificity and PPV were good for Type 2A cases in the NEE and LEE groups. On the other hand, the sensitivity before the lecture was low in both groups. By examining the misdiagnosed cases, it was found that most misdiagnoses were Type 2B. As shown in the misdiagnosis example in Additional file 1: Fig. 4, although the surface pattern is regular, the cases wherein vessels in the intervening part have various appearances or the vessel pattern is partially obscured because of the angle of incidence were likely to be misdiagnosed as Type 2B.

A majority of cases misdiagnosed as Type 2B were Type 3, whereas a majority of cases misdiagnosed as Type 3 were Type 2B. A lesion was diagnosed as Type 3 if at least one of the criteria terms in the Type 3 column in the chart was met; however, judgment may be difficult in terms of the boundary analysis between Type 2B and 3 as in the example shown in Additional file 1: Fig. 5. On the other hand, the specificity in type 3 was 90% or more in both the NEE and LEE groups before and after the lecture, which was considered to contribute to the prevention of unnecessary surgery.

We suggest that development of widely available educational tools that take care of the pitfalls clarified in this study will contribute to the improvement of diagnostic ability of endoscopists using JNET classification in facilities without specialists. Recently, endocytoscopy (Olympus, Tokyo, Japan) was developed and its high diagnostic ability has been reported [26, 27]. However, the special equipment, the knowledge and experience of endoscopists are required for the procedures and diagnoses for colorectal lesions. In addition, there have been several studies on computer-aided diagnoses that predict pathological diagnosis of colorectal lesions by NBI magnifying endoscopy [28–32]; however, sufficient accuracy of differentiation for the depth of tumor invasion has not been achieved and further research is needed. Therefore, it is considered that improvement in the diagnostic ability of endoscopists by imparting educational lectures for generalized interpretation of JNET classification will play an important role for the time being.

This study has some limitations. First, it is a single-center study, and the members of the HEE group may have been involved in obtaining and diagnosing images that were used for the NBI diagnosis tests, which may have affected the diagnostic ability. Second, as the cases were consecutive, there were differences in the number of images of each type. However, it can be considered that the data reflect actual clinical practice. Third, since all experiments were performed in one day, we have not evaluated the diagnostic ability days later after the lecture. However, we did not give the correct answer for the first test to all diagnosticians, and there was a five-hour interval between the lecture and the second NBI diagnosis test.

In conclusion, a short lecture was found to be useful for improving the diagnostic accuracy of endoscopists using JNET classification even in inexperienced or less experienced diagnosticians. Further improvement of the lecture tool is expected and may contribute to the improvement of diagnostic ability and generalization of JNET classification all over the world.

## Supplementary Information

**Additional file 1: Fig. 3**. Cases where JNET Type1 was misdiagnosed as Type 2A or 2B. a: The vessels surrounding the normal crypt are visible; however, the surface pattern is uniform honeycomb-like with regular dark spots. b: The vessel pattern is hardly visible and the normal crypts show white or dark spots. c: Isolated lacy vessels are seen and the vessels surrounding the normal crypt are visible; however, the surface pattern is uniform honeycomb-like with regular dark spots. d: The vessels surrounding the normal crypt and the isolated lacy vessels are partially visible. JNET: Japan NBI expert team. **Fig. 4**. Cases where JNET Type 2A was misdiagnosed as Type 2B. a-c: Lesions with various vessel patterns (not regular) are seen; however, a pit-like structure with smooth margin and regular structure (regular surface pattern) is seen. d, e: The edge of the vessel is irregular and partially disrupted; however, the pit-like structure is regular (regular surface pattern). JNET: Japan NBI expert team. **Fig. 5**. Misdiagnosis in cases of JNET Type 2B and 3. a: A case of Type 2B misdiagnosed as Type 3. Variable-caliber vessels and pit-like structure (the irregular surface pattern) are present. b: A case of Type 3 misdiagnosed as Type 2B. The vessels show irregular margins and distribution, also, disrupted. On the other hand, the surface structure is amorphous. JNET: Japan NBI expert team.

## Data Availability

All data generated or analyzed during this study are included in this published article and its supplementary information files.

## Abbreviations

NBI: Narrow-band imaging
JNET: Japan Narrow-Band Imaging Expert Team
NEE: No endoscopy experience
LEE: Less experienced endoscopists
HEE: Highly experienced endoscopists
NICE: NBI International Colorectal Endoscopic
HP: Hyperplastic polyp
SSP: Sessile serrated polyp
LGD: Low grade dysplasia
HGD: High grade dysplasia
SM-s: Superficial submucosal invasive
SM-d: Deep submucosal invasive
NPV: Negative predictive value
PPV: Positive predictive value
