# Supplementary material for: Effect of educational lecture on the diagnostic accuracy of Japan NBI Expert Team classification for colorectal lesions
Source: BMC Gastroenterol. 2021 Mar 5;21:110. doi: 10.1186/s12876-021-01676-x (PMC7934459; doi:10.1186/s12876-021-01676-x)
Supplement: Supplementary file 1 — Additional file 1: Fig. 3. Cases where JNET Type1 was misdiagnosed as Type 2A or 2B. a: The vessels surrounding the normal crypt are visible; however, the surface pattern is uniform honeycomb-like with regular dark spots. b: The vessel pattern is hardly visible and the normal crypts show white or dark spots. c: Isolated lacy vessels are seen and the vessels surrounding the normal crypt are visible; however, the surface pattern is uniform honeycomb-like with regular dark spots. d: The vessels surrounding the normal crypt and the isolated lacy vessels are partially visible. JNET: Japan NBI expert team. Fig. 4. Cases where JNET Type 2A was misdiagnosed as Type 2B. a-c: Lesions with various vessel patterns (not regular) are seen; however, a pit-like structure with smooth margin and regular structure (regular surface pattern) is seen. d, e: The edge of the vessel is irregular and partially disrupted; however, the pit-like structure is regular (regular surface pattern). JNET: Japan NBI expert team. Fig. 5. Misdiagnosis in cases of JNET Type 2B and 3. a: A case of Type 2B misdiagnosed as Type 3. Variable-caliber vessels and pit-like structure (the irregular surface pattern) are present. b: A case of Type 3 misdiagnosed as Type 2B. The vessels show irregular margins and distribution, also, disrupted. On the other hand, the surface structure is amorphous. JNET: Japan NBI expert team. [file 12876_2021_1676_MOESM1_ESM.docx]

***Effect of educational lecture on the diagnostic accuracy of Japan NBI Expert Team classification for colorectal lesions***

Yuki Okamoto^a^, Shiro Oka^a^, Shinji Tanaka^b^, Yuki Kamigaichi^a^, Hirosato Tamari^a^, Yasutsugu Shimohara^a^, Tomoyuki Nishimura^a^, Katsuaki Inagaki^a^, Hidenori Tanaka^a^, Kenta Matsumoto^a^, Ken Yamashita^b^, Kyoku Sumimoto^b^, Yuki Ninomiya^b^, Nana Hayashi^b^, Yasuhiko Kitadai^c^, Kenichi Yoshimura^d^, Kazuaki Chayama^a^

^a^Department of Gastroenterology and Metabolism, Hiroshima University Hospital, Hiroshima, Japan

^b^Department of Endoscopy, Hiroshima University Hospital, Hiroshima, Japan

^c^Department of Health Sciences, Prefectural University of Hiroshima, Hiroshima, Japan

^d^Center for Integrated Medical Research, Hiroshima University Hospital, Hiroshima, Japan

Short title: Effect of education on Japan NBI expert team classification

Corresponding author**:**

Shiro Oka, MD, PhD

Department of Gastroenterology and Metabolism

Hiroshima University Hospital

1-2-3, Kasumi, Minami-ku

Hiroshima 734-8551, Japan

Tel: 81-82-257-5939

Fax: 81-82-253-5939

E-mail: [oka4683@hiroshima-u.ac.jp](mailto:oka4683@hiroshima-u.ac.jp)

**Fig. 3** Cases where JNET Type 1 was misdiagnosed as Type 2A or 2B


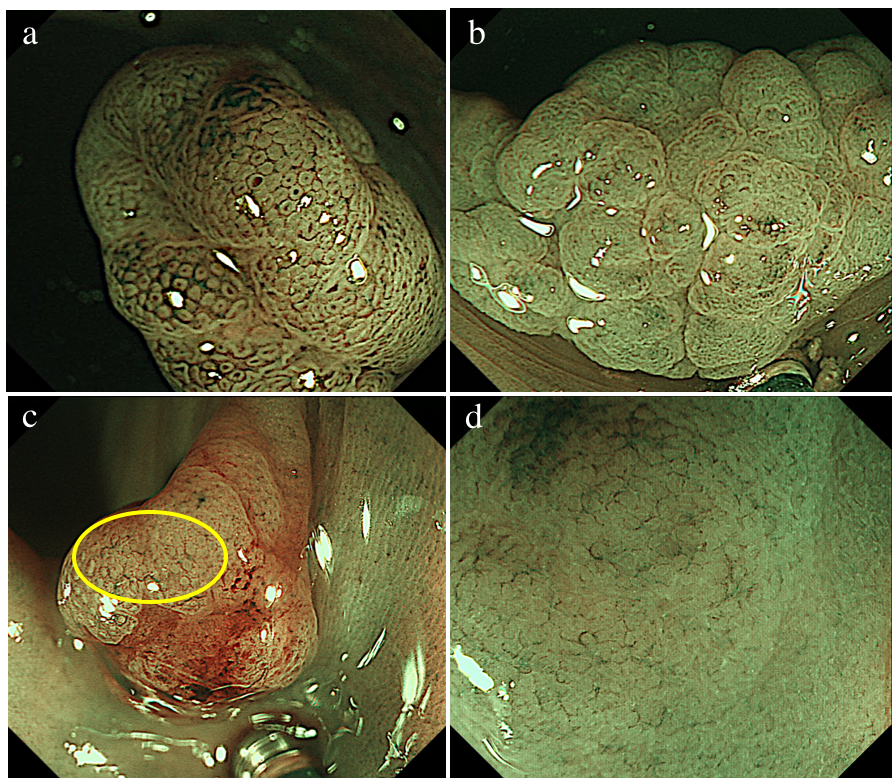


a: The vessels surrounding the normal crypt are visible; however, the surface pattern is uniform honeycomb-like with regular dark spots.

b: The vessel pattern is hardly visible and the normal crypts show white or dark spots.

c: Isolated lacy vessels are seen and the vessels surrounding the normal crypt are visible; however, the surface pattern is uniform honeycomb-like with regular dark spots.

d: The vessels surrounding the normal crypt and the isolated lacy vessels are partially visible.

JNET: Japan NBI expert team

**Fig. 4** Cases where JNET Type 2A was misdiagnosed as Type 2B


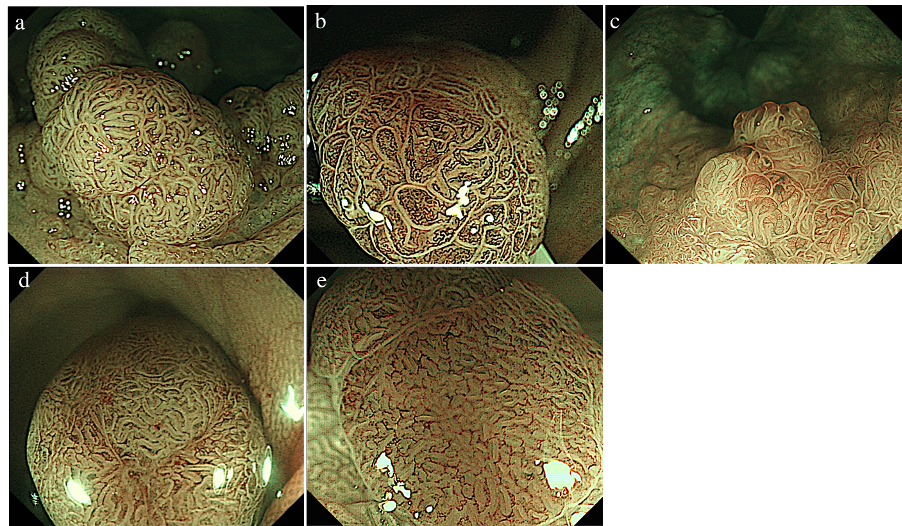


a-c: Lesions with various vessel patterns (not regular) are seen; however, a pit-like structure with smooth margin and regular structure (regular surface pattern) is seen.

d, e: The edge of the vessel is irregular and partially disrupted; however, the pit-like structure is regular (regular surface pattern).

JNET: Japan NBI expert team

**Fig. 5** Misdiagnosis in cases of JNET Type 2B and 3


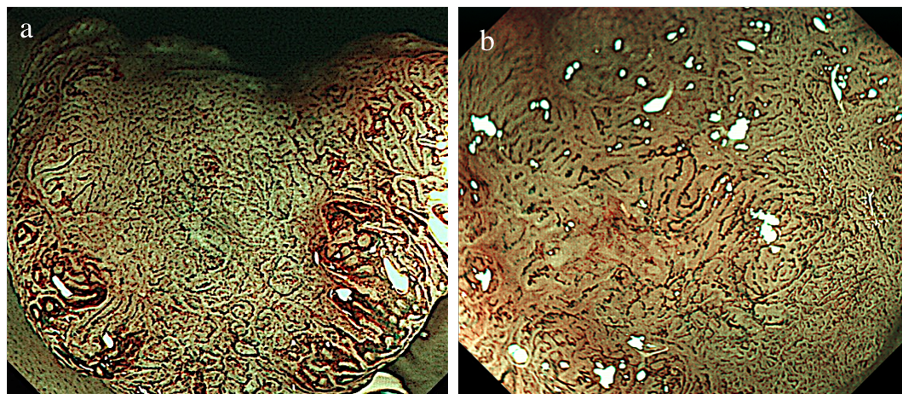


a: A case of Type 2B misdiagnosed as Type 3. Variable-caliber vessels and pit-like structure (the irregular surface pattern) are present.

b: A case of Type 3 misdiagnosed as Type 2B. The vessels show irregular margins and distribution, also, disrupted. On the other hand, the surface structure is amorphous.

JNET: Japan NBI expert team
